# An Open Source Simulation Model for Soil and Sediment Bioturbation

**DOI:** 10.1371/journal.pone.0028028

**Published:** 2011-12-05

**Authors:** Katja Schiffers, Lorna Rachel Teal, Justin Mark John Travis, Martin Solan

**Affiliations:** 1 School of Biological Sciences, University of Aberdeen, Aberdeen, United Kingdom; 2 Oceanlab, University of Aberdeen, Newburgh, Aberdeenshire, United Kingdom; National Institute of Water & Atmospheric Research, New Zealand

## Abstract

Bioturbation is one of the most widespread forms of ecological engineering and has significant implications for the structure and functioning of ecosystems, yet our understanding of the processes involved in biotic mixing remains incomplete. One reason is that, despite their value and utility, most mathematical models currently applied to bioturbation data tend to neglect aspects of the natural complexity of bioturbation in favour of mathematical simplicity. At the same time, the abstract nature of these approaches limits the application of such models to a limited range of users. Here, we contend that a movement towards process-based modelling can improve both the representation of the mechanistic basis of bioturbation and the intuitiveness of modelling approaches. In support of this initiative, we present an open source modelling framework that explicitly simulates particle displacement and a worked example to facilitate application and further development. The framework combines the advantages of rule-based lattice models with the application of parameterisable probability density functions to generate mixing on the lattice. Model parameters can be fitted by experimental data and describe particle displacement at the spatial and temporal scales at which bioturbation data is routinely collected. By using the same model structure across species, but generating species-specific parameters, a generic understanding of species-specific bioturbation behaviour can be achieved. An application to a case study and comparison with a commonly used model attest the predictive power of the approach.

## Introduction

The activities of burrowing organisms affect most, if not all, parts of the Earth's surface [1], [2]. As ecosystem engineers, they play an influential role in the structure and functioning of terrestrial, freshwater and marine ecosystems, including biogeochemical cycling and net carbon storage. Despite recognition of the importance of bioturbation over a century ago [3], resolving the mechanistic basis of how biotic activity affects soil or sediment functionality remains a challenge for contemporary ecologists. Whilst terrestrial contributions have remained largely descriptive [2], an extensive body of literature has emerged from marine benthic systems that seek to quantify the rate and spatial extent of infaunal-mediated particle and pore water fluid redistribution [4]. The principal way in which quantification has been achieved has been through the empirical administering and recovery of particulate tracers [5]–[11] following a short incubation (typically 1 d, e.g. [12], to 1 mo, e.g. [13]) in the presence of a known species or assemblage. A vertical profile of the redistributed tracer (typically at 0.5 or 1 cm resolution [14]) is then constructed and various mathematical models [15] can be fitted to the measured profile.

The most widely applied model to describe patterns of tracer profiles is the diffusion model, which applies Fick's Law of diffusion to simulate particle dispersal by analogy with diffusive heat transport and calculates a biodiffusion coefficient, Db [16]–[19]. Db is defined as the rate at which the variance of particle location changes over time, where the variance is a measure of the spread of particles in a tracer profile and is proportional to the squared velocity of the diffusing particle [20]. In recognition that species do not necessarily relocate particles diffusively, the foundation of this approach has been extended to a family of non-local models [19], [21], [22] that describe alternative modes of particle reworking reflecting observations of species-specific behaviours that translocate particles from one location to a non-adjacent location, i.e. the behaviour of epifauna (e.g. *Hyas araneus*, [23]), surficial modifiers (e.g. *Brissposis lyrifera*, [24]), gallery biodiffusers (e.g. *Hediste diversicolor*, [22]), upward (e.g. *Molpadia oolitica*, [25]) and downward conveyors (e.g. *Cirriformia grandis*, [26]) and regenerators (e.g. *Uca Pugnax*, [27]). In order to describe these different modes of particle redistribution, non-local models incorporate an exchange function K that describes particle exchange between non-adjacent sediment layers, the form of which is often specific to particular modes of particle movement [28]. Whilst such models are of great value for a mathematically coherent and elegant description of sediment particle dynamics, they are limited in providing an understanding of the ecological processes that underpin particle displacement. For example, an inherent property of the biodiffusion model is that it assumes an infinite speed of propagation, which means the model predicts tracer particles will penetrate deeper into the sediment than is physically realistic [29]. Also the mismatch between the basic assumptions of continuous mixing in differential models and distinct mixing events can, in reality, lead to a bias towards larger Db values, such that the relative contribution of infaunal bioturbation will be overestimated (see e.g. [30]). Further, the *a priori* assumptions made about how particles may be transported (e.g. [23]–[27]), although intuitive, do not necessarily account for the full suite of organism behaviour that may be encountered over time [31].

An alternative to analytical approaches is the use of stochastic, process-based simulations. Within ecology, the use of simulation models has rapidly increased [32]–[34] due to the availability of high performance computers. Random walk and lattice automaton models [35]–[39], which allow the stochastic behaviour of individual particles to be extrapolated into a deterministic description of bulk sediment transport [15], [35], have been offered as an alternative to differential models. However, these models have not been implemented in a way that allows them to be parameterised with experimental data, nor has the code been made available to facilitate testing and further development.

Recently, non-invasive imaging techniques have been developed that are capable of visualising optically distinct tracers (luminophores) at high spatial (µm) resolution over time (minutes, e.g. [23], [40], [41]), enabling the extent and influence of discrete infaunal bouts of activity on particle displacement to be quantified. Despite the step change in the quality of data these techniques provide, it remains difficult to describe key general processes with sufficiently few model parameters. The lack of such a broadly applicable solution for these high resolved data has been highlighted as a major impediment in research capability [4], [14], because the inability to establish generality limits the development of theory and replication in multiple systems [42], [43]. Here, a simulation model is presented, together with the source code and instructions on application (see worked example in Supplemental Information S1), that combines the advantages of rule-based lattice models with those of parameterisable probability density functions to generate mixing on the lattice. Our objectives are to 1) provide and demonstrate a mechanistic modelling framework that can be widely adopted and applied, and that generates ecologically relevant model output parameters that are amenable to statistical analysis and have the potential to be incorporated into ecological studies, and 2) show the applicability and predictive power of that framework using an example of highly spatio-temporally resolved experimental data on the bioturbation activity of the polychaete *Hediste diversicolor* and 3) encourage further development of a tractable framework that will hasten generic understanding through widespread application.

## Methods

### Bioturbation model

We have developed a process-based, spatially explicit (2D) simulation model that encapsulates particle displacement due to bioturbation at high temporal and spatial resolution (see worked example, *Supplemental Information S1*; sample data, *Data S1*; and programming code, *Code S1*). The core of the model represents a random walk approach for active particle movement and a discrete and stochastic version of an advection model accounting for indirect displacement ensuring mass balance across the sediment profile. In addition to these core features, the model can be adapted to account for limiting depth of particle mixture, unequal probabilities of upwards- vs. downwards movement, as well as differences in movement characteristics of marked (e.g. luminophore tracers, [44]) and non-marked particles.

The model consists of two parts. First, the active displacement of particles is simulated using a stochastic process that follows a strict set of rules (see below) defining the probability, direction and distance that each particle is displaced. Second, the model accounts for the secondary passive rearrangement of particles that must occur following any active redistribution of particles. For each time step of the simulation, the two parts of the model are repeated to determine the distribution of luminophores.

#### Model rules

The sediment is simulated as a grid of *d* rows ( = depth) and *w* columns ( = width) of cells with a side length that can be adapted to the spatial resolution of the experimental data. The *d_lum_* uppermost horizontal sediment layers represent the depth of the experimentally applied luminophores. For each luminophore pixel, the probability of being displaced is given by the constant parameter ‘*activity*’. Since the parameter ‘activity’ is negatively correlated to the rest period, the expected rest period of a particle can be calculated as (1-*activity*)×length of one time step. Each displaced particle is moved by a number of layers defined by the parameter ‘*distance*’. The direction of vertical particle displacement is drawn from a Bernoulli trial that can be parameterised as appropriate (using the parameter ‘*downwards*’) depending on the expected probabilities that particles will move either upwards or downwards. Further a limiting depth of the particle reworking activity can be set by the parameter ‘*range*’, in case information on the maximal residing depth of organisms (or particle displacement) is available.

Based on this information, particles are subtracted from layer *h_i_* and added to layers *h_i+dist_* within the sediment profile, delimited by the sediment-water interface at the upper boundary and the maximal depth, *d*, at the lower boundary. We assume wall boundary conditions since they closely reflect the natural system (i.e. particles will remain in the uppermost layer instead of being absorbed or reflected).

The active displacement of particles in one layer results in the translocation of an equivalent number of particles into a new layer, since each layer has a finite capacity defined by the width of the grid, *w*. The method which we applied to redistribute the particles accordingly is as follows: Starting from the bottom layer, the number of surplus particles is calculated. The particles to be moved are chosen randomly and relocated upwards to the layer above. To account for any differences in the characteristics of tracer and sediment particles, a weighting factor ‘*tracerdif*’ can be applied to adapt the probability of tracer displacement relative to non-marked particles. Now the same procedure is repeated for the layer above and so on until the surface of the sediment is reached. In each step, the particles that are relocated are newly chosen from all particles present in that layer. Thus, the whole upwards movement is divided into a large number of small steps by different particles. In case this procedure results in a surplus of particles at the upper-most layer (i.e. if there was active upwards-movement) the procedure is inversed and particles are step-wise moved downwards starting with the top-most layer.

#### Model parameterisation

The model described above includes three parameters (all constant in space and time) that can be estimated using high resolution data typically generated from bioturbation experiments, e.g. [23], [40], [41]: the probability of each particle to be displaced at a given time step, ‘*activity*’, the mean distance a particle is displaced ‘*distance*’, and the weighting factor ‘*tracer.dif*’ which accounts for possible differences in the dislocation probability between the tracer (luminophores) and non-marked sediment particles. The source of such particle discrimination may reflect a number of effects, including differences in the composition or surface properties of individual particles or selective particle redistribution by fauna, but is not necessarily known. Since the resolution of the model can be adapted to the resolution of the data, model simulations and experimental results can directly be compared.

To quantify the quality of the parameter values and to search for their best combination, an objective function that reflects the differences between simulation results to experimental data is needed. Here, we use the summed squares of differences (sum of sq) between the data and the model prediction for the number of luminophores in each layer and time step. The optimal combination of parameter values for the parameters is found using optimisation techniques implemented within the ‘optim’ function in the core package of the programming language R [45]. To reduce computing time, optimisation is achieved in two steps. First, a simulated annealing approach (SANN) is used to broadly approximate the global minimum of the objective function across parameter space. Whilst simulated annealing is very useful to find good parameter values on a rough surface, and has a low risk of becoming trapped at local minima [46], the method is relatively slow [47]. Thus, as a second step, the local, but faster, Broyden-Fletcher-Goldfarb-Shanno (BFGS) optimization algorithm [48]–[51] is applied to refine the optimal parameter values.

We applied the model framework described above to investigate the bioturbation activity of the ragworm *Hediste diversicolor*. In the following, we describe the experimental data, model fitting, and sensitivity analysis of the parameter values.

#### Experimental design and data collection

Sediment and individuals of the polychaete *Hediste (Nereis) diversicolor* were collected from the Ythan estuary, Scotland (57°20.085′N, 02°0.206′W). Sediment was sieved (500 µm mesh) in a seawater bath to remove macrofauna, allowed to settle for 24 h (to retain the fine fraction, <63 µm) and homogenised. Sediment was added to thin aquaria (20×5×40 cm) to a depth of 15±1 cm, overlain by 25 cm of seawater (UV sterilized, 10 µm filtered, salinity 33). Biomass was fixed at 2.0 g per aquarium (equivalent to 200 g m^−2^), a level consistent with that of the study site. Aquaria were aerated and maintained in a constant temperature room (11±2°C).

Particle bioturbation was visualised using a custom-built, time-lapse sediment profile imaging camera (f-SPI, [23]) and fluorescent-dyed sediment particles (luminophores, 125–250 µm, [44]). The f-SPI was housed inside a custom built UV illuminated imaging box (32×87×62 cm; Figure S1) consisting of a camera (Canon 400D, 3900×2600 pixels, i.e. 10.1 megapixels, effective resolution = 67×67 µm per pixel) and a UV light source (1× Phillips blacklight, 8W). The UV light source is necessary for luminophore excitation (λ = 375 to 500 nm) and provides sufficient light to illuminate the sediment profile and distinguish the sediment-water interface. Following [23], a yellow filter (Medium yellow #010, Lee Filters, UK) was fitted to the camera lens to remove light wavelengths solely used for luminophore excitation (λ = 375 to 480 nm) whilst allowing remaining light wavelengths (λ = 480 to 500 nm and λ = 700 to 800) through to the camera. The camera was set for an exposure of 10 s, f = 4.0, film speed equivalent to ISO 200 and was controlled using third party timelapse software (GB Timelapse, v. 2.0.20.0, available from http://www.granitebaysoftware.com). After an acclimatisation period of 24 h to allow macrofaunal burrow establishment, luminophores (pink, 125–250 µm, 5 g aquaria^−1^) were evenly distributed across the sediment surface before the start of the time lapse sequence. For the purpose of developing the model and reducing computing time, but also because short-term particle displacement will largely determine the displacement profile, 100 images were taken at 5 minute intervals (total experimental time = 500 mins). Observations were taken in the dark at a time that matched the natural dark period.

#### Image analysis

Images were saved in red-green-blue (RGB) colour with JPEG (Joint Photographic Experts Group) compression and analysed using a custom-made, semi-automated macro adapted from Solan et al. (2004 [23]) within ImageJ (v. 1.40), a java-based public domain program developed at the USA National Institutes of Health (available at http://rsb.info.nih.gov/ij/index.html). The user manually draws in the sediment-water interface on each image and selects an appropriate threshold to select all luminophores. As the primary interest is the vertical distribution of particles relative to the sediment water interface, it is important that depth is measured relative to the sediment-water interface. Therefore, the macro returns a binary matrix (0 = sediment, 1 = luminophores) using the sediment-water interface as the uppermost horizontal row. The total luminophores in each pixel row are then summed to provide a row total, which is used to construct the vertical profile of luminophore pixels.

#### Fitting the bioturbation model

Following the size of the experimental setup and the resolution of the image analysis, we simulated the particle displacement on a grid containing 149 vertical layers (1 layer = 1 pixel row) by 2980 pixels (horizontal width, 1 pixel = 73×73 µm). In this experiment, the applied layer of luminophore particles at time zero occupied the uppermost 20 of the 149 layers. Since we had no information on whether particles are preferentially displaced in a particular vertical direction (i.e. upwards or downwards), we assumed a symmetric distribution with a mean displacement of zero (i.e. we chose a value of 0.5 for the parameter ‘*downwards*’). The parameter ‘*range*’ was set to 1 allowing bioturbation across the whole depth of the simulated profile. With this setup we simulated 24 time steps (5 min time step^−1^).

To get a first rough estimation of the shape of the objective function across parameter space, we evaluated the sum of squares between experimental data and model predictions for a set of 512 parameter combinations. This preliminary analysis revealed a strong correlation between the parameter activity and the mean distance of particle displacement when considering their influence in the evolving sediment profile over time (Figure S2). We therefore fixed the parameter ‘*activity*’ to the best value found by the parameter scanning ( = 0.674) in order to allow for a more stable and fine-tuned optimization of the values for the parameters ‘*distance*’ and ‘*tracerdif*’. To reduce computing time, only 1/10 of the width of the sediment in the experimental set-up was modelled for the parameter estimation (the dimension of the simulated grid was d×w/10). Since we can assume that the same mixing events may occur across the whole width of the sediment, it is valid to rescale the model results to the full width afterwards in order to directly compare model results and experimental data.

#### Sensitivity analysis

Following optimisation, we performed a sensitivity analysis for the average distance a particle is displaced and the difference in movement characteristics between marked and non-marked particles. We ran the model for all possible combinations of the values for *distance* at −30% to +30% relative to the optimized value in 12 steps with equal step-size and, for *tracerdif*, from −30% relative to the optimized value to +10% in 8 steps with equal step-size (since values for *tracerdif* are restricted between 0 and 1.0). To quantify the relevance of the two parameters to the dynamics of the simulation model, the objective function is calculated for all parameter combinations as described above. The range and step-width of parameter values was chosen arbitrarily, but proved to procure an informative picture of parameter sensitivity.

## Results

**Figure 1:**
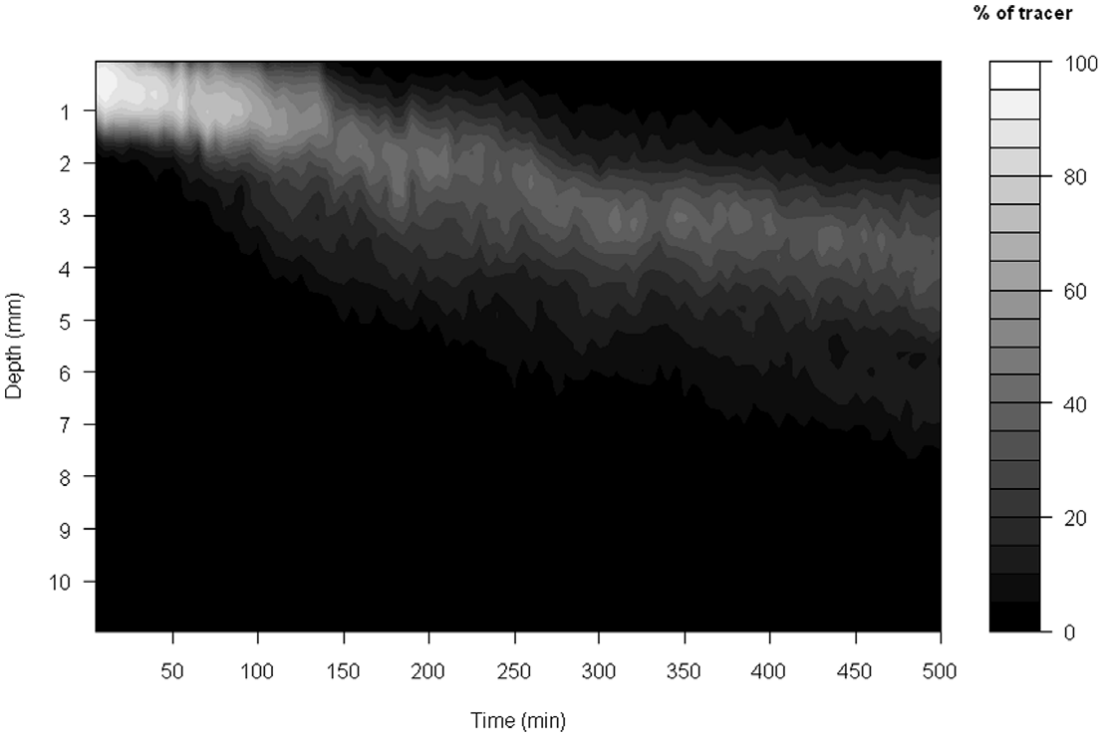
Visualisation of experimental data where grey shades denote the relative density of luminophores at a given depth (y-axis) and time point (x-axis).

The bioturbation activities of *H. diversicolor* resulted in a downward redistribution of luminophore particles over time, attaining a maximum penetration depth of ∼6 mm after 500 mins (Figure 1). During this time, individuals of *H. diversicolor* were continuously active (Sequence S1) such that the majority of the applied luminophores were located between 2–3 mm depth, but repetitive cycles of burrow relocation and construction during gallery formation translated into alternating bouts of high and low rates of particle displacement (see temporal variations in central tendency of depth trend, Figure 1). These changes, albeit subtle, were the net effect of both the upward and downward displacement of particles (e.g. at 0∶10, 0∶30 and 0∶39 s, Sequence S1), reflecting a range of frequently occurring passive and active transport mechanisms that are not necessarily analogous to exclusively diffusive or non-local descriptors.

**Figure 2:**
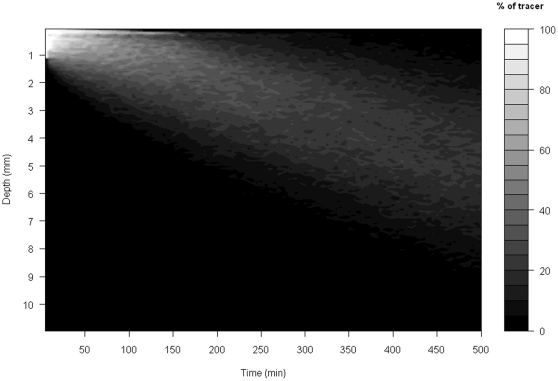
Visualisation of the results of the simulation model where grey shades denote the relative density of luminophores at a given depth (y-axis) and time point (x-axis).

By generating a visual representation of the surface of the objective function across parameter space prior to calibration (see worked example in **Supplemental Information S1**), a strong negative correlation was found between *activity* and *distance* in their effect on the spatio-temporal patterns within the bioturbation model. This meant that a wide range of value combinations (ranging from low values for *activity* and high values for *distance*, to high values of *activity* and low values for *distance*) were similarly plausible; the surface of the objective function shows a furrow rather than a clear global minimum (Figure S2). However, this preliminary optimisation showed that the best value for the parameter *activity* was 0.674. For the subsequent calibration of the two remaining parameters this value was therefore fixed to ensure a more stable optimisation procedure. The starting values for the parameter fitting using simulated annealing were *distance* = 5 and *tracerdif* = 0.9. Convergence occurred after approximately 300 iterations, returning values of *distance* = 4.242 and *tracerdif* = 0.929. Replicate (n = 10) runs of the BFGS fitting procedure indicated that the experimentally observed sediment profiles were most likely to be generated by mean particle displacements (± SD) of *distance* = 4.30±0.07 sediment layers ( = 0.314±0.03 mm), with the mean (± SD) likelihood that a non-marked particle will be dislocated upwards in the passive reallocation part of the model of 98.69±0.02%. Visualisation of the model output is depicted in Figure 2, providing a reasonable approximation of experimental observations (Figure 1).

**Figure 3:**
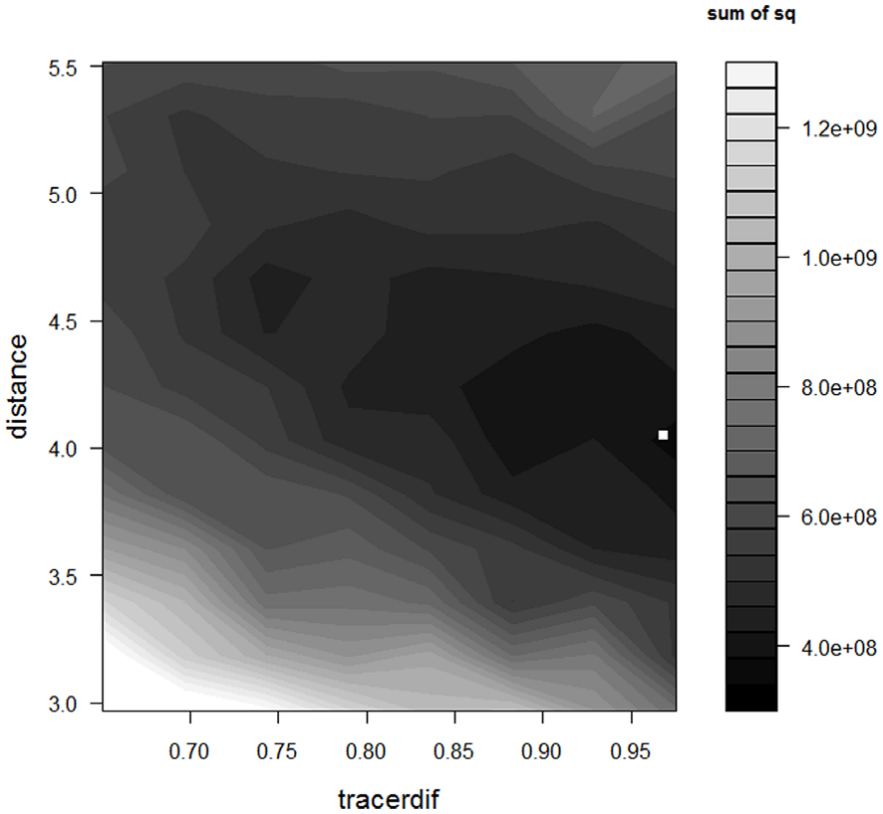
Visualisation of the sensitivity analysis (±30%) for the activity parameter and the parameter accounting for density differences between luminophores and non-marked particles, ‘*density*’. Low values of the objective function (dark grey shades) indicate a good fit between model predictions and the observed data. The white square indicates the location of the optimised parameter combination. Sum of sq = sums of squares.

Sensitivity analysis of *distance* and *tracerdif* showed a clear improvement of model predictions with rising values of *tracerdif* and mid-range values of *distance* (Figure 3). In general, however, the sensitivity of model predictions is much lower close to the global minimum (when *tracerdif*≥0.80 and *distance* is ≥4.0 but ≤5.0) relative to the edges of the parameter space.

**Figure 4:**
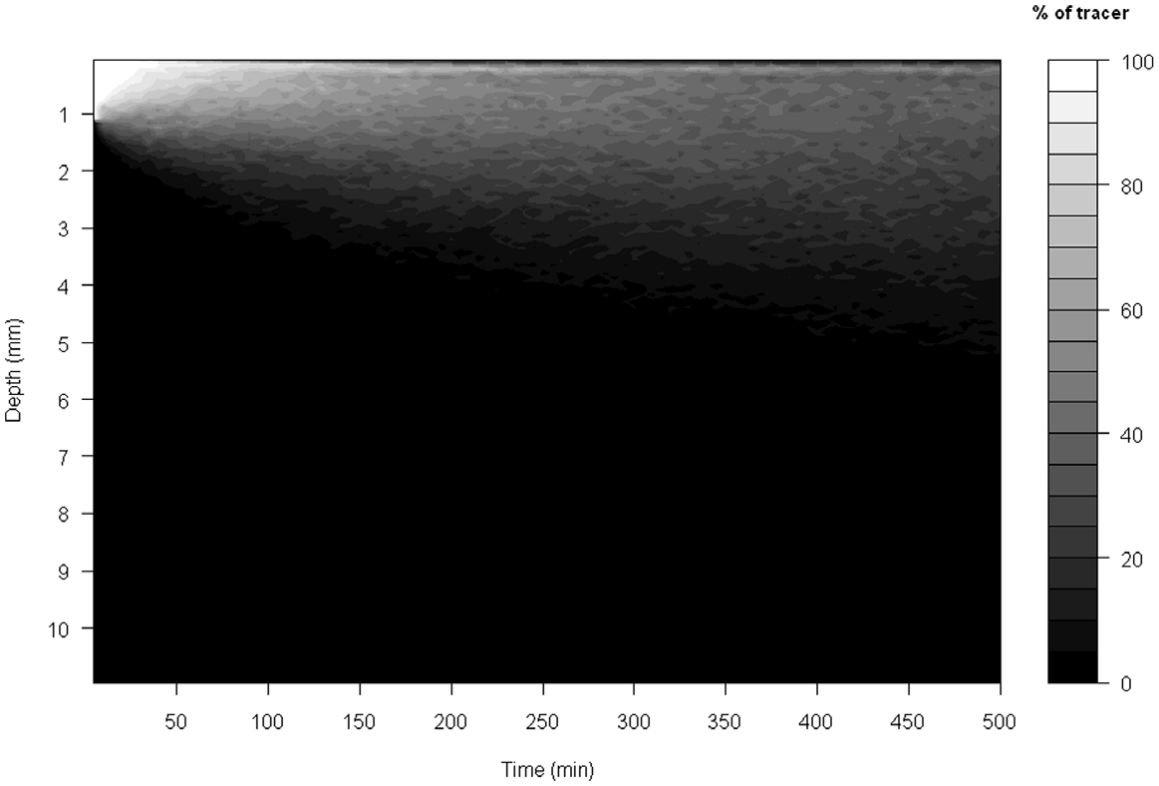
Visualisation of the predicted distribution of tracer particles (luminophores) assuming a purely diffusional form of redistribution. Grey shades denote the predicted relative density of luminophores at a given depth and time point.

**Figure 5:**
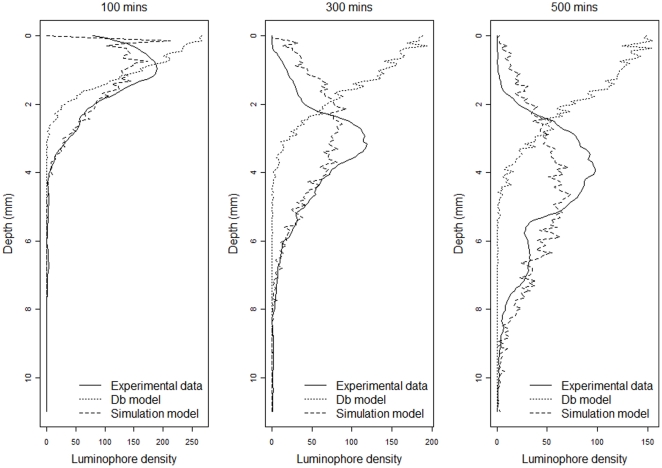
Selected profile examples of experimental data (solid line), Db predictions from a diffusional model fitted analogously to the simulation model (dotted line) and simulation model predictions (dashed line) at time t = 100 min, 300 min and 500 min. x-axis depicts number of luminophore pixels in a 300 pixel wide sediment profile.

**Figure 6:**
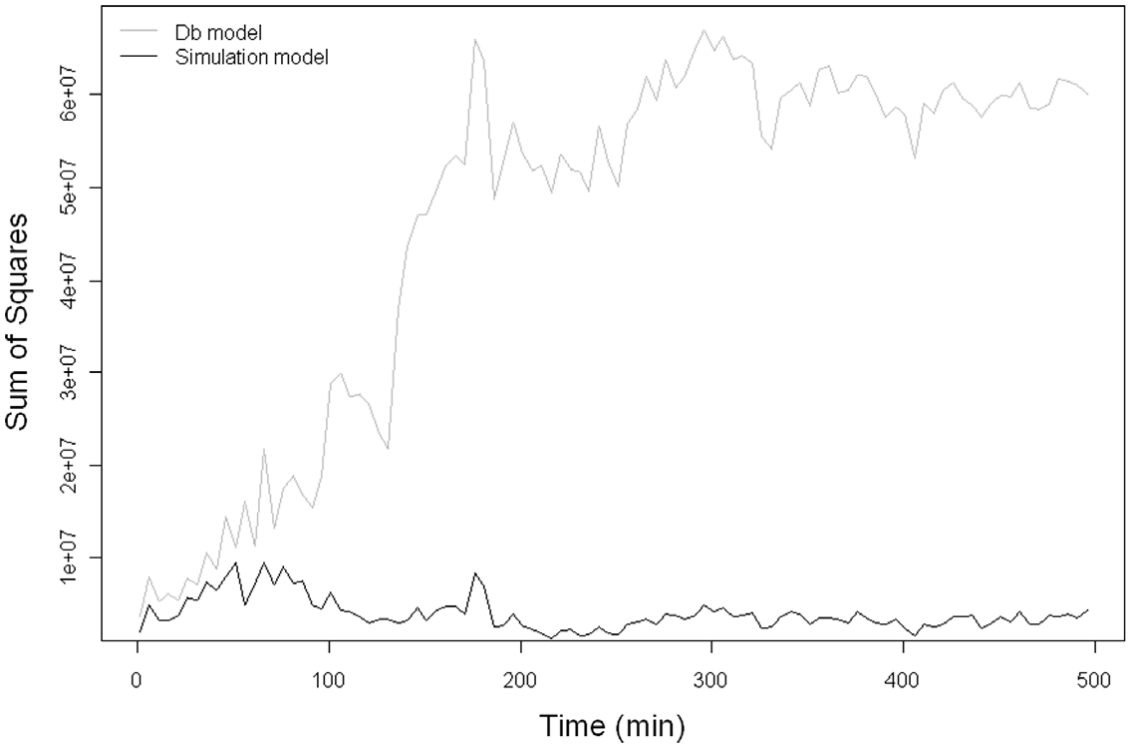
The sum of squares (measure of fit) of the commonly used Db model (grey) and the simulation model (black) over time. We acknowledge that alternative models of bioturbation (not presented here) may also show a better measure of fit than the Db model, but provide this comparison as Db is often the preferred model in empirical investigations using bioturbation as a response variable.

The capacity of the simulation model to approximate faunal-mediated particle movement is consistently better than that achieved with the version of our model assuming pure diffusion (compare model predictions in Figures 2 and 4 with the observed data in Figure 1), especially at shallower depths within the sediment profile. This improvement appears to be conserved over time (compare panels in Figure 5), even though the simulation model is describing the average movement of particles over the whole experimental time period. These data also confirm that the suitability of estimating Db decreases as luminophore profiles become more complex in shape over time and less similar to the exponential decrease described by diffusional transport. In contrast, the simulation model performs well with the sum of squares between the simulated and observed luminophore distribution pattern remaining low and less variable (Figure 6), providing confidence in the fitting procedure. Moreover, the improvement in fit of the simulation model (after ∼50 minutes) coincides with deterioration in fit of the diffusional model, providing reassurance that the simulation model is more appropriate as particle redistribution patterns become more complex and integrate a wider range of infaunal activity over time. It is important to emphasise here, however, that other bioturbation models not presented here will also show an improvement in fit over Db. Nevertheless, we make the comparison here as Db is the most frequently applied model in empirical studies that use bioturbation as a response variable [4], [52].

## Discussion

The ability to collate information on faunal mediated particle transport at high spatial (µm) and temporal (s to mins) resolution, as achieved here, has now become routine and has led to a step change in information capability on bioturbation [23], [40], [41], replacing previous methods that involved the slicing of sediment cores at low resolution (≥0.5 cm; [53]–[57]). The comparative approach we adopted here confirmed that profiles obtained at low resolution (cm) are more likely to approximate the broadly exponential decrease of tracers with depth, rather than the fine detail of the tracer distribution necessary for formulating an improved understanding of faunal mediated bioturbation. When coupled with supporting evidence from theory [15], [39] and simulation studies [30], the argument that it is no longer acceptable to model faunal mediated particle displacement at low spatio-temporal resolution becomes compelling.

As a first step towards the development of a generically applicable methodology, we have successfully applied a rule based simulation model to highly resolved spatio-temporal bioturbation data, avoiding the need for an exchange function that is specific to the mode of sediment transport [14], [22], [28]. Hence, the simulation model can be applied to a full range of infaunal species and/or assemblages and direct comparisons of the output parameters can be made using standard statistical procedures. Importantly, as strict rules define the probability, direction and distance that each particle is displaced, output parameters directly relate to the net effects of faunal reworking rather than to abstract concepts (e.g. Db refers to the rate at which the variance of particle location changes over time; [20]) that are more difficult to interpret within an ecological context.

A key objective of our model development was the inclusion of sufficient detail so that we were able to reproduce the observed distribution of tracer particles, whilst retaining sufficient simplicity that we maximised predictive power, applicability and generic value [58], [59]. Additionally, our aim was to keep the number parameters sufficiently low so that we avoided problems related to over fitting [28], [60]. Importantly, our work has revealed strong sensitivity of model output to the relative values of *distance* and *tracerdif*, indicating the potentially critical importance of accounting for differences in density or particle behaviour between marked (e.g. luminophores) and non-marked tracer particles [4]. Such tracer dependent effects occur even when the luminophores are matched as closely as possible to the natural sediment by size and has important implications for experimental design and the interpretation of tracer profiles; the simulation model was not able to obtain a subsurface peak to match experimental observations in the absence of a tracer difference (i.e. *tracerdif*). Thus, a major benefit of the modelling approach we have taken is the ease with which any differences between natural sediment and tracer particle behaviour can be identified and accounted for. Having fitted *tracerdif* to the experimental data, it is straightforward to explore (by running the model with *tracerdif* = 1.0) how tracer particles of the same density as non-marked particles would redistribute over time. A key recommendation of our work is that all future studies fitting models to similar tracer data critically evaluate whether there are differences in particle density and, where necessary, account for same.

In fitting our model to the *H. diversicolor* data, we have found there to be insufficient information to robustly fit both *activity* and *distance*. This is due to the strong negative correlation that was found between the two parameters in their effect on the spatio-temporal patterns within the bioturbation model. If our single objective was the construction of the most parsimonious model for this particular species, there would be a strong case for reducing the number of parameters from 3 to 2, collapsing *activity* and *distance* into a single parameter. However, our aim is to provide a more general framework that can incorporate species-specific or context-specific changes in infaunal behaviour where the third parameter may become necessary for explaining spatio-temporal patterns of sediment redistribution. Also, in differentiating between the likelihood of particles being displaced and the distance they are moved when displaced, retention of the two parameters promises to aid interpretation of results. Our recommendation, at least until we have sufficient information across a range of species to indicate we should do otherwise, is that the model should always be initially fitted using all three parameters.

The effectiveness of the biodiffusive model in describing the bioturbation behaviour of *H. diversicolor* has been questioned previously [13], [22], although such discussion is a distraction as alternative and more suitable models are available and investigators have not always applied Db appropriately [52]. It is important, however, to consider how the output parameters obtained here relate to the behaviour of *H. diversicolor*. It is clear that the redistribution of particles by *H. diversicolor* occurs in bouts of activity (every ∼100 minutes) that are associated with burrow construction, maintenance and the repositioning of the upper region of the burrow during the establishment of new connections with the sediment-water interface. These bouts of activity, which presumably reflect changes in feeding behaviour following resource depletion [61]–[64], result in the movement of sediment over large spatial increments (*distance* = 0.314±0.03 mm) relative to mean particle size at the study site ( = 50 µm, [62]). Importantly, advection of sediment from depth to the sediment-water interface occurs alongside the downward movement of particles (Sequence S1), highlighting that the *a priori* allocation of species to single mechanisms of particle transport (e.g. as in [22]–[27]) may not reflect changes in behaviour. By avoiding such categorisation, the output from our model is more amenable to direct comparison with other species and/or environmental contexts, as well as for correlating the faunal mediated redistribution of particles to functional measures, such as nutrient generation.

Although the model presented here considered a single species and set of circumstances, we have provided an open modelling structure that can be readily expanded and improved beyond current capabilities. We envisage that this will be particularly important as more detailed (e.g. 3- and 4-dimensional data, e.g. [65]–[66]) or more stochastic (e.g. discrete event triggered bioturbation, [23]) data becomes available in the future. Indeed, the high resolution of the data used in the present study allowed us to account for the differences in behaviour between the tracer (luminophores) and natural sediment and factor it out when characterising the species-specific parameters. Such an increased capacity for deriving more complete approximations of bioturbation is of tremendous value to, for example, efforts linking ecosystem process to changes in levels of ecosystem functioning (e.g. [57]). The modelling framework may be extended to incorporate parameters that would explicitly describe temporal variation (and indeed temporal patterns) in bioturbation activity. Such information may become particularly useful as we begin to scale-up from single to multi-species systems; when there is an assemblage of bioturbators, the spatio-temporal patterns of particle movements may be driven by a combination of, for example, high frequency local movements, (e.g. the ghost shrimp, *Neotypaea californiensis*, [67]), lower frequency (due to lower organism densities; e.g. by spider crabs, *Hyas araneus*, [23]) or periodic displacements governed by feeding behaviour (e.g snapping shrimp, *Alpheus macellarius*, [68]; bivalves, *Abra ovata* and *Abra nitida*, [41]), and/or displacement events over long distances (e.g Holothurians, *Molpadia oolitica*, [25]; Polychaetes, *Cirriformia grandis*, [26]). In the meantime, if we are to fully derive the benefit of pooling experimental efforts that attempt to formulate an improved understanding of how bioturbation contributes to global nutrient cycling, primary productivity and other components of the marine system, it is imperative that experimental replication over novelty is valued [42]–[43], and periodic reviews and meta-analyses (e.g. [4]) are undertaken with a view to applying and developing theory, establishing generality and generating predictive power that is relevant and of practical value [69]. It is our hope that the model presented here will facilitate this process.

## Supporting Information

Figure S1 **Diagram of the custom built UV illuminated imaging box showing the UV lighting (upper centre), camera (right) and aquarium (left) containing sediment (brown) and luminophores (pink).** The inside of the box is painted matt black to minimise internal reflection. A side of the box is removed in the diagram to show the inside. Drawn to scale (Box size = 32×87×62 cm).
(TIF)

Figure S2 **The sum of squares (colour shades) between the activity parameter and the mean distance of particle displacement for the sample dataset for** ***Hediste diversicolor*** **.** Tracer difference (*tracerdif*) = 0.9. The sums of squares are minimised as *distance*→6.5 and *activity*→0.5 (darkest green shading).
(TIF)

Code S1 **Programming code in Tinn-R text editor format (** http://sciviews.org/Tinn-R/ **) for the process-based, spatially explicit (2D) bioturbation simulation model.**
(R) Click here for additional data file.

Data S1 **Raw counts of the vertical distribution of pink luminophore tracer particles over time for the polychaete,** ***Hediste diversicolor*** **, used in the worked example detailed in Supplemental Information S1.**
(TXT)

Sequence S1 **Time-lapse fluorescent sediment profile imaging sequence detailing the redistribution of pink luminophore tracer particles for the polychaete,** ***Hediste diversicolor*** **.** Each frame = 5 minutes of elapsed time.
(MOV)

Supplemental Information S1 **Detailed guide on how to apply and parameterise the process-based, spatially explicit (2D) bioturbation simulation model detailed in this contribution.**
(DOC)
