# Supplementary figures and images for: An Open Source Simulation Model for Soil and Sediment Bioturbation
Source: PLoS One. 2011 Dec 5;6(12):e28028. doi: 10.1371/journal.pone.0028028 (PMC3230619; doi:10.1371/journal.pone.0028028)

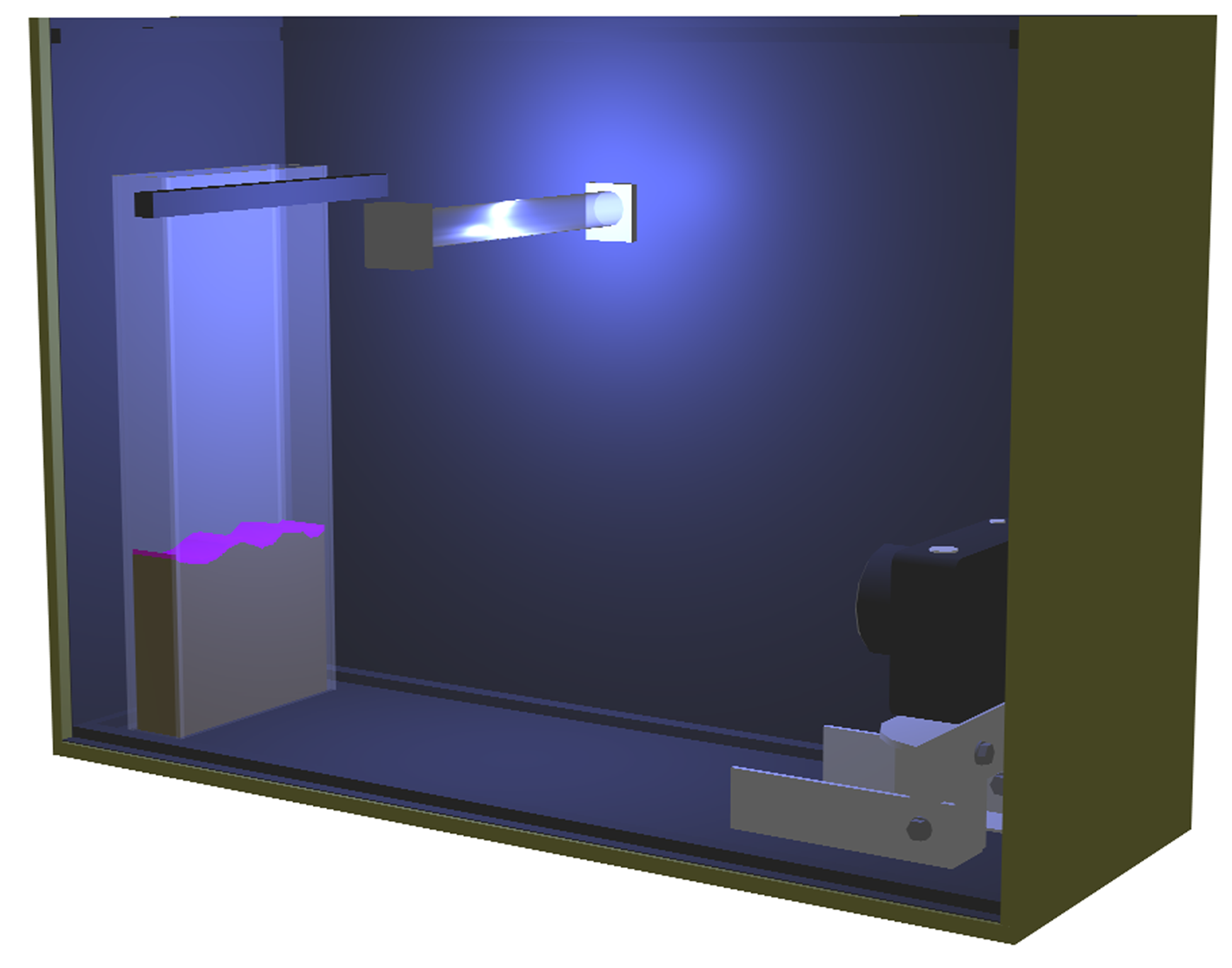

Supplement: Figure S1 — Diagram of the custom built UV illuminated imaging box showing the UV lighting (upper centre), camera (right) and aquarium (left) containing sediment (brown) and luminophores (pink). The inside of the box is painted matt black to minimise internal reflection. A side of the box is removed in the diagram to show the inside. Drawn to scale (Box size = 32×87×62 cm). (TIF) [file pone.0028028.s001.tif]

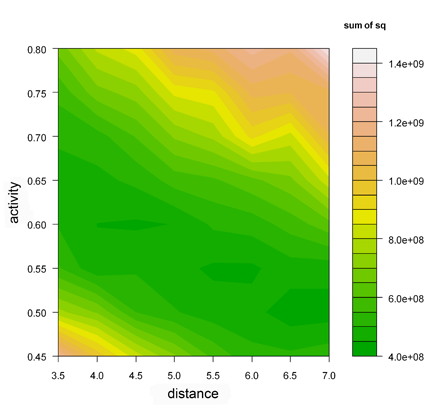

Supplement: Figure S2 — The sum of squares (colour shades) between the activity parameter and the mean distance of particle displacement for the sample dataset for Hediste diversicolor . Tracer difference (tracerdif) = 0.9. The sums of squares are minimised as distance→6.5 and activity→0.5 (darkest green shading). (TIF) [file pone.0028028.s002.tif]
