# Supplementary material for: An Open Source Simulation Model for Soil and Sediment Bioturbation
Source: PLoS One. 2011 Dec 5;6(12):e28028. doi: 10.1371/journal.pone.0028028 (PMC3230619; doi:10.1371/journal.pone.0028028)
Supplement: Supplemental Information S1 — Detailed guide on how to apply and parameterise the process-based, spatially explicit (2D) bioturbation simulation model detailed in this contribution. (DOC) [file pone.0028028.s006.doc]

**Text S1**

**Bioturbation Simulation Model (version 1.0): Worked Example**

Our bioturbation simulation model can be implemented in the open source software R. Here, we provide a step-by-step guide on how to run the model for a typical dataset (provided as *data_S1.txt*). This dataset is a simplified version of that presented in the accompanying manuscript to save computation time. Previous experience of R is not necessary. The code provided (below, and as a Tinn-R file, *code_S1.r*) has been tested using R version 2.10.1. Note that all versions of R are available from the R website (e.g. from the UK mirror site, <http://www.stats.bris.ac.uk/R/src/base/R-2/>).

**Installation of R**

1. The R package is available for most operating systems from [http://www.r-project.org](http://www.r-project.org/)
2. Click on CRAN and select an appropriate mirror site from the list provided.
3. Click on the operating system you require (Linux, Mac or Windows) and then select ‘base’.
4. Click on the download hyperlink at the top of the page and then select ‘Run’. R will then be automatically installed on your computer.

**Data format**

Our model requires a vertical distribution of a particulate tracer, such as luminophores, for a minimum of two *regularly* intervalised time points. We recommend, for most invertebrate macrofauna, that time steps should be ≤ 15 minute intervals. The vertical distribution of luminophores needs to be stored in a separate column for each time point, with the tracer count for the sediment-water interface located in the first cell and counts for deeper layers in successive cells below. Each column needs to be ordered in temporal sequence, starting with the first time point in the first column, the second time point in the second column, and so on. Each column should have a header (no spaces or special characters) in the first row (e.g. time_0_, time_1_, time_2_, time_3_…time_n_).

The data needs to be saved as a tab delimited text file (.txt). A sample dataset (*Data S1.txt*) is provided with 2159 rows and 24 columns. In this example, each row is equivalent to 0.0067 cm (i.e. one pixel dimension, = 67 μm) and each column is equivalent to 60 minutes of elapsed time (24 time points). The spatial dimension of each pixel is calculated by dividing the image width in cm by the number of pixels across the horizontal span of the image. Note that the image width may be less than the capability of the digital camera used after any extraneous detail (e.g. sides of the aquaria) are discounted from the field of view.

We provide brief explanations of each step of the process. The code that should be copied and pasted into R is provided below in red, whilst values that the user needs to specify are indicated in green or blue. Whilst it is possible to copy directly from this document, it is more convenient to copy and paste the code into a text editor such as Tinn-R, available from <http://www.sciviews.org/Tinn-R/> and provided here as *Code S1.r*.

**Step 1. Reading your data into R**

To call your data, copy and paste the following code into R:

rm(list = ls())

alldata<- read.table(file=file.choose(), header = T)

attach(alldata)

Under Windows a window will open. Under Linux you can type the path to the file you want to be used. Locate and select the *sampledata.txt* file provided.

To check the structure of the data, type:

dim(alldata)

A summary of the structure of the data will be returned confirming that you have 2159 observations (rows = ‘slices’ containing luminophores) in each of 24 columns (each column = each time point).

**Step 2. Plot the observed vertical distribution of luminophores over time**

Specify the length of each time step (in minutes), the depth of each layer or slice (in cm) of sediment that the tracer (e.g. luminophores) was recovered or imaged from (for images, a layer equates to 1 pixel row), the vertical depth of sediment (in cm) in the experimental mesocosm and the width of the region of interest (i.e. the sediment) in the image (in pixels). In this example, these are 60 minutes, 0.0067 cm, 15 cm and 2980 pixels respectively:

timestep.min <- 60

layer.cm <- 0.0067

depth.cm <- 15

sed.width <- 2980

Now it is possible to plot the data:

data <- as.matrix(alldata)

tdata <- t(data)

filled.contour(c(1:(dim(tdata)[1])), c(1:dim(tdata)[2]), ((tdata/sed.width)*100),

col=gray((0:19)/19),

levels=seq(0, 100, by=5),

xlab="Time (min)", ylab="Depth (cm)",

main="",

plot.axes = {axis(1, at = seq(1, dim(tdata)[1]+1, length.out=11),

labels = seq(0, dim(tdata)[1]* timestep.min, length.out=11))

axis(2, at = seq(from=1, to=(depth.cm/layer.cm)+1,

length.out=(depth.cm+1)),

labels = (seq(from=0, to=depth.cm, length.out=(depth.cm+1))))},

ylim=rev(range(c(1:dim(tdata)[2]))),

key.title = title(main = " % of tracer", cex.main=0.9),cex=1)

A plot of the data with time on the x-axis and sediment depth on the y-axis will be shown. The percentage of tracer will be shown in shades of white, referenced against a key as follows:

This figure (and all other figures generated throughout this document) can be copied to the clipboard or saved as an enhanced metafile (.emf) or postscript file (.ps) by right-clicking on the image (when displayed in R) and selecting the appropriate option from the drop down menu. Alternatively, you can save the figure in other formats from the File menu in the main R window.

The above image shows that at the start of the experiment (left of image) all the luminophores are at the surface (white area) and as time progresses (along the x-axis) the luminophores are incorporated deeper into the deposit (white layer moves downwards). If, as in this case, infaunal activity is restricted to layers near the surface (here, all tracer activity occurs at depths < 1 cm), you can restrict the plot so that it presents only that portion of the profile. To reduce the data matrix in this way, the first step is to specify the depth you wish to plot. In this example, 1 cm is equivalent to 149 layers (= 1 / 0.0067, i.e. 1 cm divided by the depth of each image slice in cm) which can be specified as:

alldata <- alldata[1:149,]

In order to plot the restricted data, we now repeat the plotting procedure as above with the new depth settings (i.e. depth set to 1cm and data restricted to first 149 layers) , hence:

timestep.min <- 60

layer.cm <- 0.0067

depth.cm <- 1

sed.width <- 2980

You can now plot the new restricted dataset:

data <- as.matrix(alldata)

tdata <- t(data)

filled.contour(c(1:(dim(tdata)[1])), c(1:dim(tdata)[2]), ((tdata/sed.width)*100),

col=gray((0:19)/19),

levels=seq(0, 100, by=5),

xlab="Time (min)", ylab="Depth (cm)",

main="",

plot.axes = {axis(1, at = seq(1, dim(tdata)[1]+1, length.out=11),

labels = seq(0, dim(tdata)[1]* timestep.min, length.out=11))

axis(2, at = seq(from=1, to=(depth.cm/layer.cm)+1,

length.out=(depth.cm+1)),

labels = (seq(from=0, to=depth.cm, length.out=(depth.cm+1))))},

ylim=rev(range(c(1:dim(tdata)[2]))),

key.title = title(main = " % of tracer", cex.main=0.9),cex=1)

A refined plot of the data with time on the x-axis and sediment depth on the y-axis will appear as follows:

**Step 3. Load the model**

In order to load the model, copy and paste all of the following code at into R. There are no edits necessary in this section of the code. For those familiar with modelling, we have incorporated notes into the code (lines denoted by #) that provide a brief description of what each sub-section of the code is achieving.

bioturb <- function(timesteps=timesteps, timestep.min=timestep.min, sed.width=sed.width,

depth=depth, depth.cm=depth.cm, layer=layer, layer.cm=layer.cm,

activity=activity, range=range, downwards=downwards,

distance= distance, dist.to.vari, tracerdif= tracerdif, plot=FALSE){

if(plot==TRUE & distance < 1.0){

stop("Please choose value > 1.0 for distance")

}

if(plot==TRUE & (tracerdif > 1.0 | tracerdif < 0.0)){

stop("Please choose value between 0.0 and 1.0 for tracerdif ")

}

# create vectors that represent sediment profile

sed.profile <- numeric(depth)

temp.profile <- numeric(depth)

temp.profile.m <- numeric(depth)

temp.profile.u <- numeric(depth)

diff <- numeric(depth)

sed.profile[1:layer] <- sed.width

# create matrices where results are stored

results <- matrix(0, nrow=depth, ncol=(timesteps))

results.turned <- matrix(0, nrow=(timesteps+1), ncol=depth)

results.turned[1,] <- sed.profile[1:depth]

move.prob <- c(rep(activity, range*depth), rep(0, depth-(depth*range)))

vari <- distance /dist.to.vari

######### active movement ###################

for(t in 1:timesteps){

# number of marked particles that are moved in each layer are

# drawn randomly (dep on 'move.prob') and stored in 'moved.marked'

moved.marked <- mapply(function(sed.profile, move.prob)

sum(rbinom(sed.profile, 1, move.prob)),

sed.profile, move.prob)

# same for unmarked particles

moved.unmarked <- mapply(function(sed.profile, move.prob)

sum(rbinom(sed.width-sed.profile, 1, move.prob)),

sed.profile, move.prob)

# distances for marked particles are drawn randomly (dep on 'distance' and 'vari')

distances.m <- sapply(moved.marked, function(moved.marked)

floor(rnorm(moved.marked, distance, vari)))

# same for unmarked particles

distances.u <- sapply(moved.unmarked, function(moved.unmarked)

floor(rnorm(moved.unmarked, distance, vari)))

# directions (up or down) are drawn randomly (dep on 'downwards')

directions.m <- sapply(moved.marked, function(moved.marked)

rbinom(moved.marked, 1, downwards))

# same for unmarked

directions.u <- sapply(moved.unmarked, function(moved.unmarked)

rbinom(moved.unmarked, 1, downwards))

# moved particles are substracted from temp.profiles

temp.profile.m <- temp.profile.m - moved.marked

temp.profile.u <- temp.profile.u - moved.unmarked

# and added at new layers

for(d in 1:depth){

if(length(directions.m[[d]])>0){

dest.m <- mapply(function(directions, distances)

if(directions == 1)return(d+distances)

else return(d-distances),

directions.m[[d]], distances.m[[d]])

dest.m[dest.m < 1] <- 1

dest.m[dest.m > depth] <- depth

for(i in dest.m) temp.profile.m[i] <- temp.profile.m[i] + 1

}

if(length(directions.u[[d]]) > 0){

dest.u <- mapply(function(directions, distances)

if(directions == 1) return(d+distances)

else return(d-distances),

directions.u[[d]], distances.u[[d]])

dest.u[dest.u < 1] <- 1

dest.u[dest.u > depth] <- depth

for(i in dest.u) temp.profile.u[i] <- temp.profile.u[i] + 1

}

} # layers

########### rearranging #############

# due to active movement of particles the number of particles in each layer are

# not the same. In this part of the model, the particles rearrange so that

# in each layer there are as many particles as the sediment width allows

temp.profile <- temp.profile.u + temp.profile.m

# move all particles that are too many in the respective layer one layer up

# start from the bottom

for(d in depth:2){

if(temp.profile[d] > 0){

if((sed.profile[d] + temp.profile.m[d]) > 0){

np <- max(((sed.profile[d] + temp.profile.m[d]) - sed.width),

(min((sed.profile[d] + temp.profile.m[d]),

sum(rbinom(temp.profile[d], 1,

(min(sed.width, (sed.profile[d] + temp.profile.m[d]))/sed.width)*

(1- tracerdif))))))

}

else np <- 0

temp.profile[d-1] <- temp.profile[d-1] + temp.profile[d]

temp.profile[d] <- 0

temp.profile.m[d-1] <- temp.profile.m[d-1] + np

temp.profile.m[d] <- temp.profile.m[d] - np

}

}

# move all particles that are too many in respective layer one layer down

# start from the top

for(d in 1:(depth-1)){

if(temp.profile[d] > 0){

if((sed.profile[d]+temp.profile.m[d]) > 0){

np <- max(((sed.profile[d]+temp.profile.m[d])-sed.width),

(min((sed.profile[d] + temp.profile.m[d]),

sum(rbinom(temp.profile[d], 1,

min(1.0, ((min(sed.width,

(sed.profile[d]+temp.profile.m[d]))/sed.width)

*(1+ tracerdif))))))))

}

else np <- 0

temp.profile[d+1] <- temp.profile[d+1] + temp.profile[d]

temp.profile[d] <- 0

temp.profile.m[d+1] <- temp.profile.m[d+1] + np

temp.profile.m[d] <- temp.profile.m[d] - np

}

}

###### update profiles ###########################

sed.profile <- sed.profile + temp.profile.m

if(max(sed.profile > sed.width)) cat("Sum ", sum(sed.profile))

temp.profile.m <- temp.profile.m * 0

temp.profile.u <- temp.profile.u * 0

results[,t] <- sed.profile

results.turned[(t+1),] <- sed.profile[1:depth]

# if you want to print timesteps on the screen remove hash in next line

#if(t%%10 == 0) cat("timestep ", t, "\n")

} # time steps

results.turned <- (results.turned/sed.width)*100

if(plot==TRUE){

# if plot shall be saved delete hashes in next line and before dev.off()

# postscript("model_results.eps", paper="special", horizontal= FALSE, onefile=FALSE, width=9, height=6)

filled.contour(c(1:(timesteps+1)), c(1:depth),results.turned, col=gray((0:19)/19),

levels=seq(0,100, by=5),

xlab="time (min)", ylab="depth (cm)",

main="",

plot.axes = {axis(1, at = seq(1, dim(tdata)[1]+1, length.out=11),

labels = seq(0, dim(tdata)[1]* timestep.min, length.out=11))

axis(2, at = seq(from=1, to=(depth.cm/layer.cm)+1,

length.out=(depth.cm+1)),

labels = (seq(from=0, to=depth.cm, length.out=(depth.cm+1))))},

ylim=rev(range(c(1:dim(tdata)[2]))),

key.title = title(main="% lumin"),

cex=1.5)

# dev.off()

}

}

**Step 4. Definition of the starting point for Model Parameterisation**

To reduce computation time, before full model optimisation can take place it is important to specify sensible staring points for a number of parameters involved in the model. Note that it is only necessary to run the model for the vertical extent of bioturbation that has been observed in the experimental mesocosm (see depth and depth.cm parameters below). Thus:

timesteps = the number of time steps in the time-lapse sequence (in this worked example, 24 time points)

timestep.min = the length (in minutes) of each time step (in this worked example, 60 minutes)

depth = the number of rows (layers) in your data (in this worked example, 2159 rows, but you can reduce to 149 [see step 2])

depth.cm = the depth of the sediment in the experimental mesocosm in cm that you are modelling (i.e. 1 cm if using the restricted data)

layer = the number of rows (layers) of the tracer (luminophores) at the start of the experiment (this is equivalent to the maximum depth of

luminophores at time zero, in this worked example = 20)

layer.cm = the depth of each pixel (or row) layer in cm (in this worked example, 0.0067 cm)

range = should be set to 1. This means that the whole vertical profile can be bioturbated.

downwards = should be set to 0.5. This means that the probability of each particle being displaced upwards to a higher layer within the vertical profile is the same as the probability of it being displaced downwards to a lower layer within the vertical profile.

dist.to.vari = this describes the relation between *distance* and the variance of this distance. The value is not parameterised, but we got good results using a value of 4.

sed.width = the maximum number of tracer particles possible in one layer. This is equivalent to the number of vertical columns in your data (i.e. the width of the image in pixels), in this case 2980. To minimise simulation time, this figure should be divided by 10. If you wish to simulate the whole width of the sediment do not divide by 10 in the sum.of.squares function below.

plot = this is a Boolean parameter (i.e. set to TRUE or FALSE) which determines whether results are plotted or not. Set to TRUE.

It is possible to get a rough approximation of what constitutes an appropriate starting value by manually changing the model parameters and visually comparing the observed data generated earlier with the resulting model output plot. This is achieved by altering the output parameters *distance*, *activity* and *tracerdif* alongside the parameters we have just set above. The values that delineate the output parameters are as follows:

*distance* = the average number of layers a particle is displaced in one time step, should be an integer value > 0.

*activity* = the probability that each tracer particle will be moved in one timestep. The value must be set between 0.0 and 1.0.

*tracerdif* = this sets the probability of particles being displaced in the rearrangement part of the model according to the density difference between the tracer particles and the natural sediment (non-tracer) particles. The value should lie between 0.0 and 1.0.

Now we put the last two steps together and play around with the model by changing the output parameters a few times until you are satisfied that you have selected a combination that gives good model results (by visually comparing the plot of the observed data generated earlier (in step 2) with the model output plot generated during this step). This is achieved by running the following code (values in red do not need to be adjusted, values in green reflect the input data specified under step 4, whilst the blue text are the values that need to be adjusted at each iteration until you are satisfied that you have selected a combination that returns a reasonable model approximation (generated here) of the observed data (generated in step 2):

bioturb(timesteps=24,

timestep.min=60,

depth=149,

depth.cm=1,

layer= 20,

layer.cm=0.0067,

range=1.0,

downwards=0.5,

dist.to.vari=4,

sed.width=298,

distance =8,

activity=0.5,

tracerdif =0.9,

plot=TRUE)

This will return a plot similar to the following depending on which value for *distance* and *tracerdif* you opt for:

You now need to compare the above figure to the plot of the real data generated earlier in Step 2. The aim is that the above image approximates the observed data (Step 2). It is not essential that you repeat this step many times (< 5-10 iterations are usually sufficient) as this step only provides a rough starting point for the formal parameterisation. However, the closer the match between the observed data and this output, the less computation time will be necessary in the following steps.

**5. Testing for correlations between parameters**

We now check whether the parameters *distance* and *activity* are strongly correlated so that different combinations of them result in comparably good matches between model results and observed data. If we find a strong correlation, this means that there is no definitive combination of parameter values given the observed patterns of particle redistribution and that we have to fix one of them. Note that completion of this step can take up to an hour or more depending on the amount of data you have and the specifications of your computer. First we load the bioturb_param function:

bioturb_param <- function(timesteps=timesteps, sed.width=sed.width, depth=depth, layer=layer, activity=activity, range=range, downwards=downwards, distance = distance, dist.to.vari, tracerdif = tracerdif){

if(distance < 1.0){

stop("Please choose value > 1.0 for distance")

}

if(tracerdif > 1.0 | tracerdif < 0.0){

stop("Please choose value between 0.0 and 1.0 for tracerdif ")

}

if(activity < 0.0 | activity > 1.0){

stop("Please choose value between 0.0 and 1.0 for activity")

}

# create vectors that represent sediment profile

sed.profile <- numeric(depth)

temp.profile <- numeric(depth)

temp.profile.m <- numeric(depth)

temp.profile.u <- numeric(depth)

diff <- numeric(depth)

sed.profile[1:layer] <- sed.width

# create matrices where results are stored

results <- matrix(0, nrow=depth, ncol=(timesteps))

results.turned <- matrix(0, nrow=(timesteps+1), ncol=depth)

results.turned[1,] <- sed.profile[1:depth]

move.prob <- c(rep(activity, range*depth), rep(0, depth-(depth*range)))

vari <- distance /dist.to.vari

######### active movement ###################

for(t in 1:timesteps){

# number of marked particles that are moved in each layer are

# drawn randomly (dep on 'move.prob') and stored in 'moved.marked'

moved.marked <- mapply(function(sed.profile, move.prob)

sum(rbinom(sed.profile, 1, move.prob)),

sed.profile, move.prob)

# same for unmarked particles

moved.unmarked <- mapply(function(sed.profile, move.prob)

sum(rbinom(sed.width-sed.profile, 1, move.prob)),

sed.profile, move.prob)

# distances for marked particles are drawn randomly (dep on 'distance' and 'vari')

distances.m <- sapply(moved.marked, function(moved.marked)

floor(rnorm(moved.marked, distance, vari)))

# same for unmarked particles

distances.u <- sapply(moved.unmarked, function(moved.unmarked)

floor(rnorm(moved.unmarked, distance, vari)))

# directions (up or down) are drawn randomly (dep on 'downwards')

directions.m <- sapply(moved.marked, function(moved.marked)

rbinom(moved.marked, 1, downwards))

# same for unmarked

directions.u <- sapply(moved.unmarked, function(moved.unmarked)

rbinom(moved.unmarked, 1, downwards))

# moved particles are substracted from temp.profiles

temp.profile.m <- temp.profile.m - moved.marked

temp.profile.u <- temp.profile.u - moved.unmarked

# and added at new layers

for(d in 1:depth){

if(length(directions.m[[d]])>0){

dest.m <- mapply(function(directions, distances)

if(directions == 1)return(d+distances)

else return(d-distances),

directions.m[[d]], distances.m[[d]])

dest.m[dest.m < 1] <- 1

dest.m[dest.m > depth] <- depth

for(i in dest.m) temp.profile.m[i] <- temp.profile.m[i] + 1

}

if(length(directions.u[[d]]) > 0){

dest.u <- mapply(function(directions, distances)

if(directions == 1) return(d+distances)

else return(d-distances),

directions.u[[d]], distances.u[[d]])

dest.u[dest.u < 1] <- 1

dest.u[dest.u > depth] <- depth

for(i in dest.u) temp.profile.u[i] <- temp.profile.u[i] + 1

}

} # layers

########### rearranging #############

# due to active movement of particles the number of particles in each layer are

# not the same. In this part of the model, the particles rearrange so that

# in each layer there are as many particles as the sediment width allows

temp.profile <- temp.profile.u + temp.profile.m

# move all particles that are too many in the respective layer one layer up

# start from the bottom

for(d in depth:2){

if(temp.profile[d] > 0){

if((sed.profile[d] + temp.profile.m[d]) > 0){

np <- max(((sed.profile[d] + temp.profile.m[d]) - sed.width),

(min((sed.profile[d] + temp.profile.m[d]),

sum(rbinom(temp.profile[d], 1,

(min(sed.width, (sed.profile[d] + temp.profile.m[d]))/sed.width)*

(1- tracerdif))))))

}

else np <- 0

temp.profile[d-1] <- temp.profile[d-1] + temp.profile[d]

temp.profile[d] <- 0

temp.profile.m[d-1] <- temp.profile.m[d-1] + np

temp.profile.m[d] <- temp.profile.m[d] - np

}

}

# move all particles that are too many in respective layer one layer down

# start from the top

for(d in 1:(depth-1)){

if(temp.profile[d] > 0){

if((sed.profile[d]+temp.profile.m[d]) > 0){

np <- max(((sed.profile[d]+temp.profile.m[d])-sed.width),

(min((sed.profile[d] + temp.profile.m[d]),

sum(rbinom(temp.profile[d], 1,

min(1.0, ((min(sed.width,

(sed.profile[d]+temp.profile.m[d]))/sed.width)

*(1+ tracerdif))))))))

}

else np <- 0

temp.profile[d+1] <- temp.profile[d+1] + temp.profile[d]

temp.profile[d] <- 0

temp.profile.m[d+1] <- temp.profile.m[d+1] + np

temp.profile.m[d] <- temp.profile.m[d] - np

}

}

###### update profiles ###########################

sed.profile <- sed.profile + temp.profile.m

if(max(sed.profile > sed.width)) cat("Sum ", sum(sed.profile))

temp.profile.m <- temp.profile.m * 0

temp.profile.u <- temp.profile.u * 0

results[,t] <- sed.profile

## if you want to print timesteps on the screen remove hash in next line

# if(t%%10 == 0) cat("timestep ", t, "\n")

} # time steps

return(results)

}

Next we define an objective function that calculates the quality of the match between the observed results and the model output. Here we use the sum of squared differences between the number of particles in each layer and each time step:

sum.of.squares_three <- function(par, obs, sim.rep, sim.depth, sim.timesteps, ...){

if(par[1] < 1.0 || par[2] <= 0.0 || par[2] > 1.0 || par[3] <= 0.0 || par[3] > 1.0){

return(NA)

}

else{

obs <- (obs[1:sim.depth, 1:sim.timesteps])

pre <- array(0, c(sim.depth, sim.timesteps))

for(t in 1:sim.rep){

pre <- try(pre+(bioturb_param(distance =par[1], activity=par[2], tracerdif =par[3],

depth=sim.depth, timesteps=sim.timesteps, ...)))

}

sos <- sum((obs-(pre/sim.rep))*(obs-(pre/sim.rep)))

return(sos)

}

}

Next, we decide on a parameter range of *distance* and *activity* that we want to test and the step width. Here we calculate the objective function for all possible combinations of values from 11.0 to 14.5 in steps of 0.5 for the *distance* and values from 0.6 to 0.95 in steps of 0.05 for *activity*. The parameter *tracerdif* is kept constant at a value that resulted from step 4 (the exact value is not crucial since it will not significantly influence the correlation pattern between the two tested parameters). First, we need a matrix to store the results of the analysis. For each parameter value of *distance,* one row, and for each value of *activity,* one column.

Remember to change the following (in green and blue text) as appropriate:

results <- matrix(0, ncol=8, nrow=8)

x <- 1

y <- 1

for(md in seq(from=11.0, to=14.5, by=0.5)){

for(a in seq(from=0.6, to=0.95, by=0.05)){

pars <- c(distance =md, activity=a, tracerdif =0.9)

sos <- sum.of.squares_three(par=pars, obs=alldata, sim.rep=1, sim.depth=149, sim.timesteps=24,

sed.width=298, layer=20, range=1.0, downwards=0.5,

dist.to.vari=4)

results[x,y] <- sos

x <- x+1

}

y <- y+1

x <- 1

}

You can plot the results of this step by entering:

(Note that the first two rows should contain the same sequences of parameter values as used above)

filled.contour(seq(from=11.0, to=14.5, by=0.5),

seq(from=0.6, to=0.95, by=0.05),

results,

col=terrain.colors(28),

xlab="distance", ylab="activity",

main="",

cex.lab=1.0,

key.title = title(main = "sum of sq", cex.main=0.9))

It will look something like the following:

If the plot reveals a diagonal of low values for the sum of squares (rather than a central area of low values) it means that several combinations of the two tested parameters result in a good match between model output and experimental results and that these parameters are correlated. In this case, go to step 6, otherwise go directly to step 7. In the above plot, a green diagonal line would clearly mean correlation, but here it shows that the optimal values might be located outside of the parameter range we have tested. To be optimal (no correlation), there would be a green centre somewhere within the confines of the plot indicating the area where the sum of squares are low. Note that this plot may have to be redrawn with extended scales on the x- and y-axes to incorporate the optimal location where the sums of squares are minimised.

**6. Model Parameterisation with correlated parameters *activity* and *distance***

Model parameterisation is achieved in two steps: simulated annealing, which is known to provide good rough estimates of optimal parameter values, followed by the Broyden Fletcher Goldfarb Shanno (BFGS) method to achieve finer resolution of results. Use the parameter combination that gave the best visual comparison between the plot of the model output and the plot of the observed data (i.e. the output parameters marked in blue in step 4) as the starting values for the optimization procedure. This is specified by entering:

pars <- c(distance=8, tracerdif =0.9)

obs <- alldata

Next we need a version of the objective function with only two variable parameters:

sum.of.squares_two <- function(par, obs, sim.rep, sim.depth, sim.timesteps, ...){

if(par[1] < 1.0 || par[2] <= 0.0 || par[2] > 1.0){

return(NA)

}

else{

obs <- (obs[1:sim.depth, 1:sim.timesteps])

pre <- array(0, c(sim.depth, sim.timesteps))

for(t in 1:sim.rep){

pre <- try(pre+(bioturb_param(distance=par[1], tracerdif =par[2],

depth=sim.depth, timesteps=sim.timesteps, ...)))

}

sos <- sum((obs-(pre/sim.rep))*(obs-(pre/sim.rep)))

return(sos)

}

}

Before you can start the simulated annealing process, you need to enter the parameter values determined in step 4. In the code that follows:

sim.depth = depth (as in step 4)

sim.timesteps = timesteps (as in step 4)

sed.width = sed.width (as in step 4)

layer = layer (as in step 4)

activity = 0.5 (as in step 4)

range = 1.0 (as in step 4)

downwards = 0.5 (as in step 4)

dist.to vari = 4 (as in step 4)

maxit = controls the number of iterations and is the only stopping criterion

parscale = should contain the chosen starting values determined in step 4 for *distance* and *tracerdif*. It is important that these

values are separated by a comma in the order *distance*, *tracerdif.*

Using the above guide, change the green numbers below to fit your data, match the blue numbers to those determined in step 4, and run the following code. You should also change the value for activity to something that resembles the value obtained when the sums of squares are minimised in Part 5 above (i.e. estimate the value from the correlation graph - darkest green shading). Note that you may have to redraw the correlation graph with different axes (in Part 5) to obtain a suitable value. Depending on the amount of data you have, the following process can take from minutes to days:

fitmodel_SANN <- optim(par=pars, sum.of.squares,

obs=obs, sim.rep=1, sim.depth=149, sim.timesteps=24,

sed.width=298, layer=20, activity=0.5, range=1.0, downwards=0.5,

dist.to.vari=4, plot=FALSE, method=c("SANN"),

control=list(trace = 6, parscale=c(8, 0.9),

maxit=1000))

The above process goes through 999 iterations to get a reasonable approximation of the parameters. The R console will show you that the process has started by printing (values can vary):

sann objective function values

initial value 557095677.000000

With the sampledata provided this process can take up to three hours (dependant on computer power). The SANN will most likely **not** converge and therefore result in an error message that will alert you to this effect. You will see the following appear in your R console (values can vary):

iter 999 value 520027161.000000

final value 520027161.000000

sann stopped after 999 iterations

This is normal and can be ignored. The simulated annealing process is a means to provide a good rough estimate of the optimal parameter values before a final resolution methodology is employed. Convergence is therefore achieved in the next step (BFGS).

To show the results of the simulated annealing process, enter:

fitmodel_SANN

The output should look like this:

$par

distance tracerdif

20.0587646 0.4836768

$value

[1] 520027161

$counts

function gradient

1000 NA

$convergence

[1] 0

$message

NULL

Note that the values for *distance* and *tracerdif* (in blue) will differ each time the SANN is run as these are not the definitive final parameter values but a close approximation.

Now specify the revised parameter values fitted by SANN *from the above output* as follows (i.e. the blue values are determined by the previous output):

pars <- c(distance = 20.0587646 , tracerdif = 0.4836768)

Before you can start the BFGS fitting process, you need to enter the parameter values determined by the simulated annealing process. Adapt the parameter values using the new parameters (can be rounded to reduce decimal places) in the code below (blue text), check that the other parameters reflect those from step 4 (green text), and then copy and paste the code into R to start BFGS fitting:

fitmodel_BFGS <- optim(par=pars, sum.of.squares,

obs=obs,sim.rep=1,sim.depth=149, sim.timesteps=24,

sed.width=298, layer=20, activity=0.5, range=1.0, downwards=0.5,

dist.to.vari=4, plot=FALSE, method=c("BFGS"),

control=list(trace = 6, parscale=c(20.06,0.48),

maxit=10000, ndeps=c(0.01,0.001)))

The BFGS process **must converge to achieve the best fit for the parameters**. The code above sets the maximum number of iterations to 10000. This continues until the model converges, at which point you will see:

initial value 522414787.000000

final value 520112673.000000

converged

If this convergence does not occur within the given maximum of 10000 iterations, the parameters can be tweaked in the BFGS fitting code (last two lines of last step). Specifically, the maximum number of iterations can be increased (maxit = *xxxxx*, where *xxxxx* is >10000) or the ndeps values can be increased to maximise the region of convergence, i.e. it is easier for the model to converge, but the best fit output is slightly less accurate. The ndeps values refer, in order of appearance, to the accuracy attributed to the fit for *distance* and *tracerdif* values.

**6a. Model output for model Parameterisation with correlated parameters *activity* and *distance***

Once convergence has taken place, the best fit parameters from the BFGS fitting procedure are called by typing:

fitmodel_BFGS

The output provides the **optimal parameter combination** and should look like this:

$par

distance tracerdif

21.0238424 0.8807801

$value

[1] 520112673

$counts

function gradient

152 5

$convergence

[1] 0

$message

NULL

Note again that the values for *distance* and *tracerdif* can differ each time the BFGS is run as model convergence is achieved within a certain margin. To get an idea of the variability (and stability) of the output values it is advisable to re-run the BFGS fitting procedure a number of times and calculate a mean ± SD of the model output parameters. These can be achieved after running the simulated annealing fitting procedure only once. Trials with the present data suggest that 5-10 repeats of the BFGS fitting procedure are sufficient. Hence, the final parameter values should be presented with error.

Note that the value for *distance* is returned as the number of layers or slices. To convert to the unit of measurement (cm), enter the following (where the blue text is the output value for *distance* from the previous step):

distance.cm <- 21.0238424*layer.cm

distance.cm

This will return the value for *distance* in cm.

[1] 0.1408597

Plotting of the model to compare to the observed data can be achieved by:

bioturb(timesteps=24,

timestep.min=60,

depth=149,

depth.cm=1,

layer= 20,

layer.cm=0.0067,

range=1.0,

downwards=0.5,

dist.to.vari=4,

sed.width=298,

distance=21.0238424,

activity=0.5,

tracerdif =0.8807801,

plot=TRUE)

which gives the following plot:

If the sums of squares are needed, these can be saved to file using:

model_results <- bioturb_param(timesteps=24,

depth=149,

layer= 20,

range=1.0,

downwards=0.5,

dist.to.vari=4,

sed.width=298,

distance =21.0238424,

activity=0.5,

tracerdif =0.8807801)

sum_of_squares <- colSums((obs - model_results)*(obs - model_results))

Now specify where you wish the file to be saved (modify green text below as appropriate):

write.table(sum_of_squares,"D:/directory/filename.txt", col.names=T, row.names=F)

**7. Parameterisation with uncorrelated parameters *activity* and *distance***

Model parameterisation is achieved in two steps: simulated annealing, which is known to provide good rough estimates of optimal parameter values, followed by the Broyden Fletcher Goldfarb Shanno (BFGS) method to achieve finer resolution of results. Use the parameter combination that gave the best visual comparison between the plot of the model output and the plot of the observed data (i.e. the output parameters marked in blue in step 4) as the starting values for the optimization procedure. This is specified by entering:

pars <- c(distance =8, tracerdif =0.9, activity=0.5)

obs <- alldata

Before you can start the simulated annealing process, you need to enter the parameter values determined in step 4 in the code that follows:

sim.depth = depth (as in step 4)

sim.timesteps = timesteps (as in step 4)

sed.width = sed.width (as in step 4)

layer = layer (as in step 4)

range = 1.0 (as in step 4)

downwards = 0.5 (as in step 4)

dist.to vari = 4 (as in step 4)

maxit = controls the number of replicates and is the only stopping criterion

parscale = should contain the chosen starting values determined in step 4 for *distance* and *tracerdif*. It is important that these values are separated

by a comma in the order *distance*, *activity* and *tracerdif*.

Using the above guide, change the numbers below to fit your data. Note that, depending on the amount of data you have, this process can take from minutes to days:

fitmodel_SANN <- optim(par=pars, sum.of.squares,

obs=obs, sim.rep=1, sim.depth=149, sim.timesteps=24,

sed.width=298, layer=20, range=1.0, downwards=0.5,

dist.to.vari=4, plot=FALSE, method=c("SANN"),

control=list(trace = 6, parscale=c(8, 0.9, 0.5),

maxit=1000))

The above process goes through 999 iterations to get a reasonable approximation of the parameters, but it may not converge and an error message will alert you to this effect. As the simulated annealing process is a means to provide a good rough estimate of the optimal parameter values before a final resolution methodology is employed, this can be ignored as convergence is achieved in the next step (BFGS).

To show the results of the simulated annealing process, enter:

fitmodel_SANN

The output should look like this:

$par

distance tracerdif activity

17.9780688 0.6343807 0.9345826

$value

[1] 520698433

$counts

function gradient

1000 NA

$convergence

[1] 0

$message

NULL

Now specify the revised parameter values fitted by SANN from the above output as follows:

pars <- c(distance =17.9780688, tracerdif=0.6343807, activity=0.9345826)

Before you can start the BFGS fitting process, you need to enter the parameter values determined by the simulated annealing process. Adapt the parameter values using the new parameters in the code below (blue text) and then copy and paste the code into R to start BFGS fitting:

fitmodel_BFGS <- optim(par=pars, sum.of.squares,

obs=obs,sim.rep=1,sim.depth=149, sim.timesteps=24,

sed.width=298, layer=20, range=1.0, downwards=0.5,

dist.to.vari=4, plot=FALSE, method=c("BFGS"),

control=list(trace = 6, parscale=c(17.98,0.63 ,0.93),

maxit=10000, ndeps=c(0.01,0.001,0.001)))

The BFGS process must converge to achieve the best fit for the parameters. The code above sets the maximum number of iterations to 10000. This continues until the model converges. If this convergence does not occur, the parameters can be tweaked. Specifically, the maximum number of iterations can be increased (maxit = *xxxxx*) or the ndeps values can be increased to maximise the region of convergence, i.e. it is easier for the model to converge, but the best fit output is slightly less accurate. The ndeps values refer, in order of appearance, to the accuracy attributed to the fit for *distance*, *tracerdif* and *activity* values.

**7a. Model output for parameterisation with uncorrelated parameters *activity* and *distance***

Once convergence has taken place, the best fit parameters from the BFGS fitting procedure are called by typing:

fitmodel_BFGS

The output provides the optimal parameter combination and should look as follows:

$par

distance tracerdif activity

16.0911331 0.6876334 0.9380592

$value

[1] 521412709

$counts

function gradient

128 4

$convergence

[1] 0

$message

NULL

Plotting of the model to compare to the observed data can be achieved by:

bioturb(timesteps=24,

timestep.min=60,

depth=149,

depth.cm=1,

layer= 20,

layer.cm=0.0067,

range=1.0,

downwards=0.5,

dist.to.vari=4,

sed.width=298,

distance=16.0911331,

activity=0.9380592,

tracerdif =0.6876334,

plot=TRUE)

which gives the following plot:

If the sums of squares are needed, these can be saved to file using:

model_results <- bioturb_param(timesteps=24,

depth=149,

layer= 20,

range=1.0,

downwards=0.5,

dist.to.vari=4,

sed.width=298,

distance=16.0911331,

activity=0.9380592,

tracerdif=0.6876334)

sum_of_squares <- colSums((obs - model_results)*(obs - model_results))

Now specify where you wish the file to be saved (modify green text below as appropriate):

write.table(sum_of_squares,"D:/directory/filename.txt", col.names=T, row.names=F)

**END OF WORKED EXAMPLE**

**Future development of the Bioturbation Simulation Model (v. 1.0)**

In the spirit of open source peer group collaboration, we have provided the source code and detailed explanation of our bioturbation simulation model. This model is not intended as a one-stop solution to all bioturbation modelling needs, nor does it replace alternative bioturbation models. Rather, we provide a tractable solution for empiricists and it is our hope that this model can be developed beyond what we have provided here as new information becomes available and the appropriateness of this approach is tested more thoroughly and across a wide range of contexts. An important aspect of this process (as we have done here) will be to (1) maintain and update the source code, (2) provide detailed instructions of how to run the model (and any associated analyses used during the modelling process) in such a way that a non-specialist can understand and implement the code, and (3) update and supply new code, raw data and instructions that are needed to generate additional supporting statistics or supporting graphical summaries. Our hope is that such instructions will assume no familiarity and form an instructive guide as to why certain steps are applied and how they are achieved. Guidance should also be given for when, and under what circumstances, certain components should and should not be applied. We therefore suggest that this document (Text S1) should be retained and modified and updated in future developments, i.e. it should serve as the user manual (present version referenced as Text S1 in Schiffers et al.) and be added as supplementary online material. It will also be important to maintain the development history, so we request that any future updates should reference this contribution (and any subsequent modifications by others, including publication details) at the start of this file, so that a full sequence history can be readily tracked and opportunities or limitations of the methodology can be appreciated by those applying the model to ecological data.

**END OF TEXT S1**
